# Optic Radiation Fiber Tractography in Glioma Patients Based on High Angular Resolution Diffusion Imaging with Compressed Sensing Compared with Diffusion Tensor Imaging - Initial Experience

**DOI:** 10.1371/journal.pone.0070973

**Published:** 2013-07-26

**Authors:** Daniela Kuhnt, Miriam H. A. Bauer, Jens Sommer, Dorit Merhof, Christopher Nimsky

**Affiliations:** 1 Department of Neurosurgery, University of Marburg, Marburg, Germany; 2 International Clinical Research Center, St. Anne's University Hospital Brno, Brno, Czech Republic; 3 Department of Psychiatry, University of Marburg, Marburg, Germany; 4 Institute of Imaging and Computer Vision, Rhenish-Westphalian Technical University Aachen, Aachen, Germany; 5 Department Of Mathematics and Computer Science, University of Marburg, Marburg, Germany; Charité University Medicine Berlin, Germany

## Abstract

**Objective:**

Up to now, fiber tractography in the clinical routine is mostly based on diffusion tensor imaging (DTI). However, there are known drawbacks in the resolution of crossing or kissing fibers and in the vicinity of a tumor or edema. These restrictions can be overcome by tractography based on High Angular Resolution Diffusion Imaging (HARDI) which in turn requires larger numbers of gradients resulting in longer acquisition times. Using compressed sensing (CS) techniques, HARDI signals can be obtained by using less non-collinear diffusion gradients, thus enabling the use of HARDI-based fiber tractography in the clinical routine.

**Methods:**

Eight patients with gliomas in the temporal lobe, in proximity to the optic radiation (OR), underwent 3T MRI including a diffusion-weighted dataset with 30 gradient directions. Fiber tractography of the OR using a deterministic streamline algorithm based on DTI was compared to tractography based on reconstructed diffusion signals using HARDI+CS.

**Results:**

HARDI+CS based tractography displayed the OR more conclusively compared to the DTI-based results in all eight cases. In particular, the potential of HARDI+CS-based tractography was observed for cases of high grade gliomas with significant peritumoral edema, larger tumor size or closer proximity of tumor and reconstructed fiber tract.

**Conclusions:**

Overcoming the problem of long acquisition times, HARDI+CS seems to be a promising basis for fiber tractography of the OR in regions of disturbed diffusion, areas of high interest in glioma surgery.

## Introduction

Due to their rapid growth and infiltrative nature, gliomas remain one of the challenges in neurological surgery, particularly those tumors located in direct vicinity of eloquent cortical areas or fiber bundles. Maximum extent of tumor volume resection has been accepted as the major goal in glioma surgery, being associated with a significant benefit in patient survival [1], [2], [3], whilst in any case preservation of neurological functions have to be considered. Thus, besides long-time used electrostimulation methods, particular magnetic resonance imaging (MRI)-modalities were established to display functional cortical sites or fiber bundles non-invasively. These data can be integrated into the navigation system and displayed intraoperatively, which has been shown to contribute to low postoperative morbidity [4], [5].

Due to its practicability, the estimated course and location of white matter tracts in clinical practice is currently performed mainly on the basis of diffusion tensor imaging (DTI)-MRI. DTI is based on a set of diffusion images acquired for at least six different gradient directions and one reference image [6]. With these data, the diffusion properties within each voxel can be calculated by the Stejskal Tanner equation, resulting in a 2^nd^ order tensor. In anisotropic tensors, the main eigenvector encodes for the longitudinal direction of axons in major white matter tracts. For the tractography procedure, algorithms are applied that process the tensor information and reconstruct fiber bundles. Different algorithms have already been implemented and investigated so far [7], [8], [9]. However, due to the restricted 2^nd^ order tensor model, multiple fiber populations within a voxel cannot be resolved adequately. This causes complications to resolve crossing-, kissing-, diverging or highly curved fibers. Another problem appearing with DTI-based fiber tractography is the resolution of fibers in areas of disturbed diffusion, for example due to tumor or peritumoral edema [10], [11], [12].

To overcome these drawbacks, alternative diffusion imaging and reconstruction schemes denoted as High Angular Resolution Diffusion Imaging (HARDI) [13] have become increasingly relevant. A three-dimensional orientation distribution function is herewith interpolated as a steady function on the sphere, for example by spherical harmonics. However, the clinical use of HARDI is limited due to larger number of diffusion-encoding gradients (ranging from 60 to 100), resulting in long acquisition times [14], [15], [16]. A solution for this restriction is a sparse representation of signals, which is provided for example by the use of spherical ridgelets, as proposed by Michailovich et al. [17], [18], [19].

Using this protocol, HARDI data can be represented using a relatively small number of diffusion-encoding gradients, thus enhancing the feasibility of HARDI-based fiber tractography in the clinical practice. This is implied in compressed sensing techniques (CS) [17], [20], [21], [22].

To investigate the feasibility of HARDI+CS for the reconstruction of the optic radiation (OR) in proximity of gliomas in the temporal lobe with associated disturbed diffusion properties, we performed fiber tractography based on DTI and HARDI+CS for eight patients. To our knowledge, this is the first clinical application and comparison of DTI-, and HARDI+CS-based fiber tractography for the OR, showing the potential of HARDI with respect to challenging intra-voxel fiber distributions.

## Materials and Methods

### Patients

The study was conducted as a prospective case series with retrospective data analysis after approval by the ethics commission of the Philipps-University of Marburg, Germany. All patients gave their written informed consent to participate in the study. The informed consent form was also approved by the ethics commission, Philipps-University of Marburg, Germany. Eight patients with gliomas, all located in the left temporal lobe, were included in our study. Mean patient age was 54.8 years. Three female patients and five male patients participated. To compare tumor size including peritumoral edema and their influence on the fiber reconstruction, all tumors were manually segmented across all slices. In case the tumor showed contrast-enhancement, both T1+gadolinium-, and T2-weighted tumor outlines were segmented. Otherwise, only T2-weighted tumor outlines were segmented. Tumor volumes are given in [cm^3^]. Furthermore the minimum distance from the tumor outlines to the fiber object, based on HARDI+CS was calculated in [mm]. All tumors were located in close proximity to the reconstructed OR with less than 20 mm (see Table 1).

**Table 1:** Patient Collective.

no	age	gender	lesion	minimum distance tumor/HARDI+CS-fiber [mm]	tumor volume T1+Gd/T2 [cm^3^]
1	73	m	Anaplastic oligodendroglioma WHO III	10.0	11.2/35.2
2	65	f	Glioblastoma multiforme WHO IV	18.5	10.7/68.3
3	41	m	Anaplastic astrocytoma WHO III	16.6	-/13.7
4	52	m	Glioblastoma multiforme WHO IV	10.2	10.2/16.0
5	61	f	Anaplastic astrocytoma WHO III	11.5	1.7/41.8
6	35	m	Diffuse Astrocytome WHO II	11.3	-/16.2
7	45	m	Glioblastoma multiforme WHO IV	7.6	23.7/91.1
8	66	f	Glioblastoma multiforme WHO IV	10.0	44.25/118

### MRI

MR images were acquired at a 3T MRI (Tim Trio, Siemens, Erlangen, Germany) preoperatively, including T1-weighted 3D images (3D-Magnetization Prepared Rapid Gradient Echo (MPRAGE)): repetition time (TR) 1900 ms, echo time (TE) 2.26 ms, field of view (FoV) 256 mm, matrix 256×256, slice thickness 1 mm, 176 slices, sagittal). The protocol for diffusion imaging was TR 7800 ms, TE 90 ms, FoV 256 mm, matrix 128×128, slice thickness 2 mm, numbers of excitation (NEX) = 1, b = 1000 s/mm^2^, 30 non-collinear diffusion-encoding gradients, voxel size of 2×2×2 mm^3^. This same diffusion images were the base for DTI-based fiber tractography and HARDI+CS-based fiber tractography. Acquisition of diffusion imaging data took 5 minutes per patient.

### Fiber tractography

Evaluation was performed using the image analysis platform MedAlyVis (Medical Analysis and Visualization) [23] for both, DTI-based and advanced fiber tractography using HARDI signals derived from sparsely sampled diffusion data (HARDI+CS). For DTI-based fiber tractography, a tensor deflection approach with fractional anistotropy (FA) thresholds of 0.18–0.2 was applied. For tractography based on HARDI+CS, a deterministic multidirectional orientation distribution function (ODF) tracking was applied [12] with the L-index [24] as anisotropy measure, set to 0.03 (maximum angle: 90 degrees, maximum steps: 500, stepsize: 1).

For both, DTI-, and HARDI+CS-based fiber reconstruction, the same manually segmented region around the lateral geniculate nucleus (LGN) was used as seed region. The visual cortex was then used as include region, resulting in a fiber object passing through the LGN and terminating in the visual cortex (Brodman areas 17–19). These ROIs were determined by experienced examiners (over six years of expertise), one neurosurgeon and one neurologist.

## Results – Case Series

### Case 1

A 74 year old male patient presented with a temporal tumor with slight and diffuse contrast enhancement in T1-weighted MRI. T2-weighted MR-signal however revealed mass of approximately (app.) 35 cm^3^ (Table 1).

**Figure 1:**
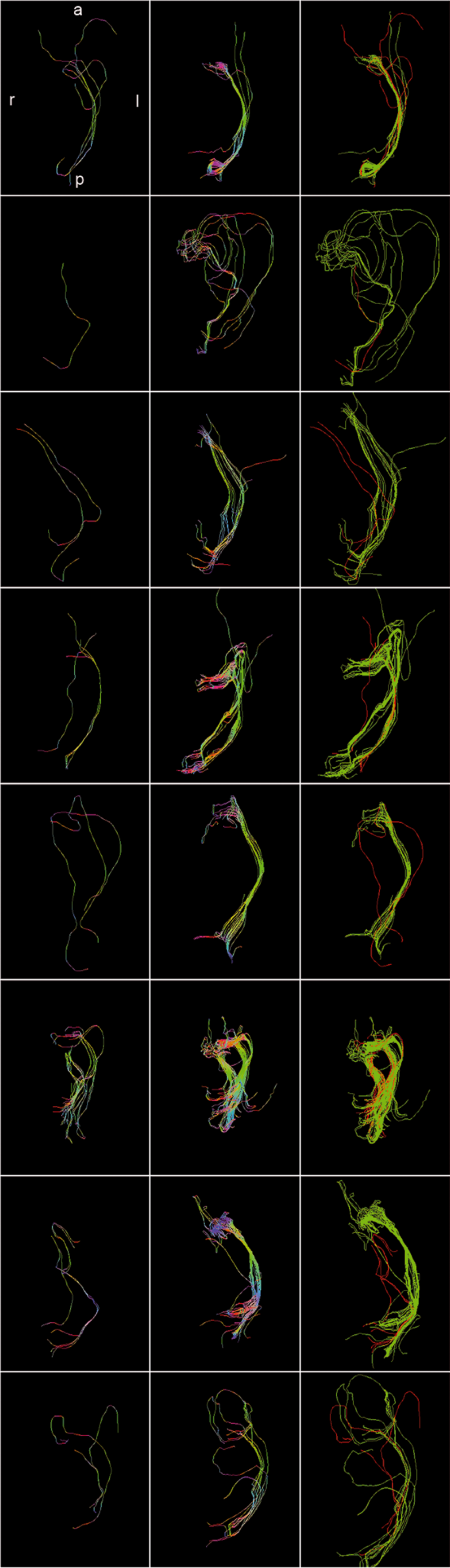
Fiber tractography results presented for each patient (patients 1–8 according rows 1–8) based on DTI (column 1), Slicer 4 (column 2), and based on HARDI+CS (column 4) within MedAlyVis. Overlay of DTI-based (red) and HARDI+CS-based tractography (green) (r = right; l = left; a = anterior; p = posterior).

For both DTI and HARDI+CS-based fiber tractography, we found a fiber bundle, reaching from LGN to the visual cortex. However, DTI-based fibers were slender and diffusely orientated. HARDI+CS-based tractography, however displayed a more solid fiber bundle, representing the OR without significant looping of Meyer's loop (ML). Still, all three parts of the OR in relation to the ventricular system are reproducible with both methods (Figure 1, row 1).

### Case 2

A 65 year-old female patient initially presented with aphasia. T1-weighted MRI scans showed a contrast enhancing tumor in the left temporal lobe of app. 11 cm^3^ with central necrosis, however with a strong peritumoral edema (Table 1).

In this case, on the basis of DTI no fiber bundle passing through LGN and terminating in the visual cortex was reliably traced. Only one single fiber was found, which can hardly be assigned definitely to one of the three parts of the OR. This is opposed to HARDI+CS-based fiber tractography, which displayed the OR with its three bundles including the ML, forming a pronounced loop in anterolateral direction (Figure 1, row 2).

### Case 3

HARDI+CS-based fiber tractography of this 41 year-old male patient with a relatively small anaplastic astrocytoma (WHO III) in the left temporal lobe, displayed a fiber bundle from LGN to the calcarine sulcus. In this case, the ML does not loop significantly. However, tractography results also include some diffusely running fibers, rather not belonging to the OR (in anterior temporo-mesial direction and directing to the corpus callosum). Single-ROI DTI-based fiber tracking results were inconclusively, with only one fiber starting from LGN and terminating in the visual cortex, most likely belonging to the central bundle of the OR (Figure 1, row 3).

### Case 4

A 52 year-old female patient was admitted with a GBM (WHO IV) of app. 10 cm^3^ and little peritumoral edema. Tumor localization is in close proximity to the OR with only app. 10 mm minimum distance. Results of DTI-, and HARDI+CS-based fiber tractography vary significantly. With HARDI+CS, the OR is reliably displayed. The ML shows a sharp loop in anterolateral direction. By contrast, using the same starting ROI around the LGN, DTI provides a slight fiber bundle, most likely showing the central bundle of the OR. The ML is not traced sufficiently (Figure 1 (row 4)).

### Case 5

A 61 year-old female patient was admitted with MRI scans of a small temporal tumor on contrast enhanced T1-weighted images, however T2-weighted images revealed a hyperintense signal of app. 42 cm^3^, closely located to the OR, given by tractography results (Table 1).

DTI-based tracking results displayed one fiber, which can each be assigned to the central, superior and inferior bundle of the OR. However, the result remains unconvincing. Compared to the HARDI+CS-based fiber bundle and contrary to the DTI-based fibers, the OR here is given as a solid bundle, particularly the ML with a sharp loop (Figure 1, row 5).

### Case 6

**Figure 2:**
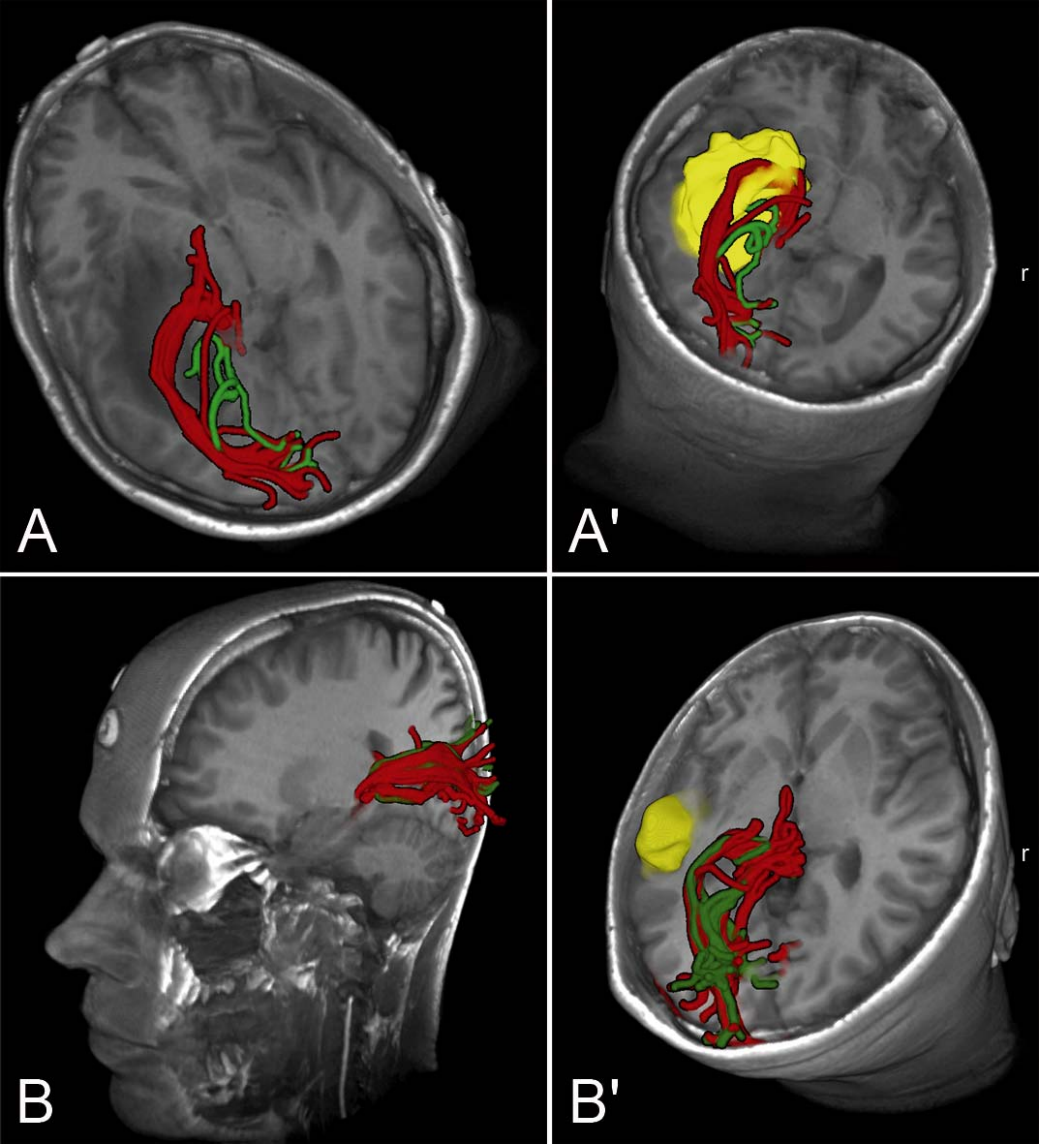
3-D models of T1-weighted MR-images with overlay of HARDI+CS-(red), and DTI-(green)-based tractography of the optic radiation presented as hulled fiber objects. A+A′: Case presentation (case 7): Case of a large temporo-mesial GBM of a 45 year-old male patient, showing the marked differences of HARDI+CS-, and DTI-based fiber objects in case of a large, high-grade tumor. A: axial oblique view. A′: Axial oblique view. Tumor manually segmented in yellow. B+B′: Case presentation (case 6): 35 year-old male patient with small diffuse astrocytoma. Less remarkable differences of tractography results in case of this smaller, low-grade tumor. B: Left sagittal oblique view. B′: Axial oblique view. Tumor manually segmented in yellow.

In a case of a 35 year-old male patient, only T2-weighted MRI images showed a small signal alteration in the lateral temporal lobe without contrast enhancement in T1-weighted images. Both single-ROI tractography results displayed the OR well with central, superior and inferior fibers, forming ML (Figure 2). However, HARDI+CS-based tractography showed a more solid and dense fiber bundle compared to DTI-based tracking. Particularly the ML, looping slightly in anterolateral direction, is displayed more convincingly by HARDI+CS-based tractography (Figure 1, row 6).

### Case 7

In this case of a 45 year old male patient with a left temporal GBM (WHO IV) accompanied with a large peritumoral edema (app. 24 and 92 cm^3^), tractography results based on DTI displayed solitary fibers, connecting LGN and visual cortex. However, fibers were not safely traced around the temporal horn of the lateral ventricle. The HARDI+CS-based results displayed a solid OR-object, showing the three bundles of the OR in their course around the temporal horn (Figure 1, row 7). Displayed within a T1-weighted 3D image model it can furthermore be seen, that the fiber bundle is significantly elevated due to the large tumor (Figure 2).

### Case 8

A 66 year old female patient presented with aphasia and hemiparesis. This corresponded to a large temporal tumor on MRI, in close vicinity to the HARDI+CS-based fiber-bundle (Table 1). This object reliably displayed the OR as a compound bundle from LGN to the visual cortex. On the basis of DTI, the OR was not displayed convincingly, as again only one single fiber can be traced from LGN to the visual cortex (Figure 1, row 8).

### Summary

Tractography results based on HARDI+CS display the OR more convincingly compared with DTI-based fiber objects in all eight cases. Overall, HARDI+CS-based objects contain more fibers and the OR is given as a more compound object. Thus, in six out of eight cases, HARDI+CS-based fiber tractography results in significantly improved fiber objects (patients 2–5, 7, 8) compared with DTI. Even in two cases (patients 2, 8), DTI based fiber tractography fails to generate an OR-object at all.

The ML is given in four cases via HARDI-based tractography, in which it can not be reliably displayed by DTI (patients 2, 4, 7, 8). In all 6 cases (patients 2–5, 7, 8), in which HARDI+CS delivered much better tractography results, patients suffered from high-grade tumors that were also associated with significant edema in 4 cases (patients 2, 5, 7, 8). Also closer location of tumor and OR (in patients 4, 5, 7, 8) resulted in significantly improved results via HARDI+CS compared with DTI.

## Discussion

### Fiber tractography of the OR and applications in clinical practice

The classical descriptions of the visual pathway, particularly the OR are based on dissection studies by Leuret and Gratiolet [25] in 1839 or Meyer [26] in 1907. These were revised and complemented with findings from newer studies [27], [28], mostly based on dissection techniques by Klingler et al. [29]. Basically, the OR consists of three bundles – anterior, central and posterior bundles (otherwise named inferior, central and superior bundles). Starting at the LGN, they pass through the temporal stem to terminate in the calcarine sulcus. In the deep white matter of the inferior limiting sulcus of the temporal lobe, they cover the superior and lateral wall of the lateral ventricle's temporal horn. Meyer [26] described the anterior bundle as the most anterior extent of the OR, looping the roof of the temporal horn behind the anterior commissure, now called ML. The central bundle crosses the roof of the temporal horn without forming an anterolateral loop. The posterior bundle instead is thought to run straight in posterior direction from the LGN in the lateral wall of the ventricle. However, the temporal stem contains multiple fiber bundles, e.g. the uncinate fascicle or the inferior occipito-frontal fascicle, so that the more recent studies found these fascicles hard to accurately delineate from each other [30]. In this way, the visual pathways still accounts for the neuroanatomically most complex fiber bundles [31].

Navigation systems are widely used among neurosurgical operating theatres, showing outlines of pre-operatively segmented risk structures and targets in the microscope heads-up display. This requires a previous registration process of physical space and image space [32]. Besides merely anatomical MR images, functional data have also been integrated in recent years, now designated as multimodality navigation. This concept includes the display of eloquent cortical sites (given for example by fMRI [33]), metabolic data (given for example by magnetic resonance spectroscopy imaging [34], single photon emission computed tomography) or major white matter tracts computed with fiber tractography.

The by far most frequently used basis for fiber tractography in the clinical practice is DTI, which is still developed further including the processes of data acquisition, image processing and analysis [35]. The principle DTI is based on the assumption that diffusion is faster alongside white matter tracts than perpendicular to the fiber bundle direction [6]. Although consisting of one b0-image and at least six non-collinear diffusion images, today a total of 30 gradient directions in a diffusion dataset has been proposed for acceptable tractography results of neuroanatomically complex fiber bundles and is thus the baseline standard for a DTI dataset [36]. One major restriction of the DTI technique is the incapability to resolve complex intra-voxel diffusion profiles such as crossing, kissing or diverging fibers. However, this is of special interest for tractography results of neuroanatomically complex fiber bundles like for example the visual pathways, particularly the OR in its course through the temporal lobe. So far, several groups have already succeeded in a DTI-based reconstruction of ML and OR [27], [37]. However their quantitative results (e.g. measured distances from temporal lobe structures like the temporal horn or the temporal pole) varied significantly [27], [38], [39]. A simiilar observation was made in studies using pre-, and postoperative visual field deficits for validation [37], [39]. A major drawback is the incapability of DTI to resolve fibers in areas of disturbed diffusion reliably, which is most commonly the case in neurosurgical patient collectives. Apart from DTI-tractography of the OR in temporal lobe epilepsy [37], in which there is no structural change of the white matter, fiber tractography is of major interest for temporal gliomas as highly invasive and infiltrative tumors are hard to delineate from the surrounding healthy brain parenchyma.

Although DTI-based tractography results have been shown to contribute to a low postoperative morbidity when integrated in the navigation system [4], [5], comparable to electrostimulation methods [40], [41], methods to provide even higher patient safety are still under intense investigation:

Apart from alternative approaches [42] or even the application of intraoperative DTI [43] using high-field intraoperative MRI systems, several approaches for improvement of the DTI-tractography procedure itself have been published. Regarding the OR, there has to be mentioned fiber tracking from multiple seed volumes as proposed by Wu et al. [44] or Tao et al. [45], variations on the number of directional motion probing gradients [38] or tractography algorithms, for example the advanced fast marching algorithm by Staempfli et al. [46] among others.

Despite these innovations, the previously described intrinsic drawbacks of the 2^nd^ order tensor model remain. These can be overcome by using advanced diffusion imaging and reconstruction schemes based on HARDI. Using this approach, multiple intravoxel fiber orientations can be resolved. However, acquisition of HARDI data sets requires a significantly higher number of diffusion gradients, ranging from 60 to 100, with associated data acquisition times of up to 25 minutes as opposed to approximately 4 minutes for DTI (on 3T MRI-systems). In this way, clinical applications of HARDI are still rare although frequently used for theoretical neuroimaging e.g. by Frey et al. [47].

The disadvantage of increased acquisition times has been alleviated by HARDI+CS, which is based on the theory of sparse representation. Thus, CS enables the reconstruction of HARDI signals from as low as 20 diffusion gradients, although with a low reconstruction error of approximately 1%. In this way, of conventional diffusion weighted data set, which is also used for DTI-based fiber tractography can be used for reconstruction [17].

Apart from this, current research also addresses higher practicability for HARDI-based fiber reconstruction in clinical applications. Due to the increased diffusion information provided by HARDI, naïve fiber tracking approaches based on HARDI data are computationally expensive. Several frameworks have been proposed, for example by Prckowska et al. [48] using a fused DTI/HARDI visualization or Reisert et al. [49].

Particularly for reconstruction of neuroanatomically complex fiber bundles, a high impact of advanced diffusion models as base for fiber tractography is likely. We investigated HARDI+CS's possible advantages over DTI on the example of the OR in a neurosurgical patient collective, all patients suffering from gliomas in the temporal lobe with their associated more complex white matter architecture.

### Interpretation of our tractography results

The basis for interpretation of our results are classical and more recently published dissection studies also mentioned above [27], [28].

In all eight cases, tractography results based on HARDI+CS display the OR better compared with DTI-based fiber objects. HARDI+CS-based objects generally display more fibers, thus displaying the OR as a solid tract including its three different bundles. This suggests, according to the recent literature that DTI-based fiber tractography generally underdetermines the extent of a fiber bundle [50].

In six out of eight cases however, HARDI+CS-based fiber tractography results in significantly improved fiber objects (patients 2–5, 7, 8) compared with DTI. In these cases, the single-ROI DTI-based results only give slender fiber bundles (patients 3–5, 7) or even do not display the OR convincingly at all (patients 2, 8).

These obvious advantages of HARDI+CS-based fiber tractography are particularly associated with the following factors:

tumor size/size of peritumoral edema
tumor localization/distance from the OR
tumor histopathology
tumor morphology in MRI

Thus, in those cases for which HARDI+CS delivered significantly better results, we found a high-grade invasive tumor in six cases (patients 2–5, 7, 8), mostly associated with a significant peritumoral edema (4 cases: patients 2, 5, 7, 8). Only one of these tumors (patient 3) did not show significant contrast enhancement on the T1-weighted MRI. Furthermore, the tumors were located in direct vicinity to the OR based on HARDI+CS with less than 15 mm in four cases (patients 4, 5, 7, 8). Compared to this, single-ROI DTI-based fiber tractography resulted in acceptable objects in two patients (1 and 6). Here, both tumors showed no contrast enhancement in T1-weighted MRI, suggesting less invasive behaviour, which was confirmed by histopathology (oligodendroglioma, diffuse astrocytoma). Furthermore, these tumors were comparatively small according to manual segmentation (35 cm^3^ and 16 cm^3^).

Comparing our DTI-based tractography results of the OR with those of other study groups, we can conclude that our results seem to be of lower quality. However, there are several explanations for this: Most DTI-based tractography results were performed in healthy brain [38], [44], [46], [51] or, if used in neurosurgical practice, in temporal lobe epilepsy without any structural changes in MRI [37]. In other cases, multi-ROI approaches were used with seed volumes placed around the LGN and alongside the course the OR [38], [44], [46], [51]. Furthermore, the FA-threshold for DTI-based tractography varies significantly or is not mentioned. In some cases a threshold as low as 0.15 [45] was described, which can lead to false estimation of the created object's volume.

To obtain comparable results for DTI-, and HARDI+CS-based fiber tractography, we used FA-thresholds of 0.18–0.2 and only one identical ROI around the LGN as start for the tractography.

### Potentials and drawbacks of HARDI+CS-based fiber tractography

As shown, we found obvious advantages of HARDI+CS-based fiber tractography over DTI-based tractography in brain white matter areas of disturbed diffusion properties. This can be of particular neurosurgical interest in high-grade glioma surgery of the temporal lobe or also for low-grade gliomas which are closely located to the OR, as DTI-based tractography of the OR seems to be of minor quality in these cases.

Of course, results of fiber tractography differ for the various intracerebral fiber bundles, so that our described findings should be investigated and ensured for other neurosurgically relevant pathways as well, particularly for those with a neuroanatomically complex course. So far, this was already done for the language-associated pathways in a glioma patient collective [52]. Here, we found similar results in terms of improved tractography based on HARDI+CS, particularly in cases of closely located larger intraaxial high-grade tumors associated with increased peritumoral edema.

However, certain drawbacks of the HARDI+CS-based fiber reconstruction have to be mentioned. Although using the same diffusion dataset with 30 non-collinear gradients, time for HARDI+CS-based fiber tractography (including the calculation of ODFs and the fiber tracking procedure itself) is significantly longer with 45 minutes, compared with 5 minutes for DTI-based tractography. In this way, clinical practicability is still reduced, requiring larger effort in time and personnel. Furthermore, the software platform MedAlyVis, on which HARDI+CS is implemented, is not commercially available and strongly emphazises scientific applications. We found, that the teach-in period is significantly longer compared with DTI-tractography applications on clinically orientated and commercially used navigation systems.

## Outlook

For optimization, we intend to vary parameters (e.g. gantry tilt, voxel size, repetitions) of the diffusion imaging sequence based for the fiber reconstruction via DTI-, and HARDI+CS. This will first be investigated with a software phantom, providing a ground truth fiber bundle to objectively compare the reconstructed fibers. Subsequently, the most promising sequence parameters will be evaluated in a collective of healthy subjects. Furthermore, optimization of tractography data for fibers with neuroanatomically complex course such the language-associated pathways, the optic radiation or the limbic system will be investigated via use of different tractography algorithms applied also to the HARDI data and compared with the tensor deflection algorithm. For future investigations, HARDI+CS-based reconstruction the mentioned fiber bundles should be routinely integrated into the navigation system besides conventional DTI-based tractography to support our hypothesis and evaluate the clinical impact on postoperative morbidity. In this context, it should be the aim to apply HARDI+CS in an open source software platform e.g. *Slicer* [53]. To implement the fiber object obtained via HARDI+CS in the intraoperatively used navigation system, a binary mask of the derived fiber bundle can be used for visualization. The intraoperatively displayed data should be compared and validated using the pre-, and postoperative neurological-, and neuropsychological examination and intraoperative electrostimulation methods, particularly subcortical stimulation. Furthermore, in case of the language-associated pathways, awake surgery can be considered in selected cases.

To provide an intraoperative fiber-estimation with compensation for brainshift, we suggest matching preoperatively obtained HARDI+CS fibers with intraoperative MRI using non-linear registration or sophisticated pattern recognition techniques [54]. Alternatively, non linear registration techniques can also be applied to other intraoperative imaging methods like 3D ultrasound, providing multimodal information intraoperatively [55], [56].

## Conclusions

With our prospectively conducted case series on eight patients, we can clearly show the potential of HARDI+CS-based fiber tractography compared with conventionally used DTI-based fiber reconstruction. HARDI+CS thus offers high resolution fiber tractography, even for neuroanatomically complex fiber bundles like the OR and in areas of disturbed diffusion patterns, however using low data acquisition times required for clinical use. In this way, a neurosurgical major interest focuses on glioma surgery of the temporal lobe, as in these cases peritumoral diffusion patterns have to be considered disturbed in any case. HARDI+CS seems to be a promising approach, combining the advantages of HARDI's estimation of multiple intravoxel fiber populations in the temporal stem with the clinical feasibility of routinely used DTI image data acquisition in presurgical practice.
